# Supplementary material for: Time from treatment initiation to HIV viral suppression in public care facilities in Brazil: A nationwide linked databases cohort
Source: PLoS One. 2024 Nov 20;19(11):e0305311. doi: 10.1371/journal.pone.0305311 (PMC11578461; doi:10.1371/journal.pone.0305311)
Supplement: S1 Table — Qualiaids-Brazil Cohort, 2015–2018 (N = 101,822). (DOCX) [file pone.0305311.s001.docx]

**S1 Table. Time between ART initiation and VL testing in the first six months of treatment^a^.** Qualiaids-Brazil Cohort, 2015-2018 (N = 101,822)

| Time from ART initiation to 1^st^ VL | Individuals | | Median time to VS (days) |
| --- | --- | --- | --- |
|  | With a test | VS achievement in the 1^st^ test |  |
| ≤66 days^b^ | 42,159 (32.8) | 10,849 (25.7) | 55.0 |
| 67-90 days | 18,010 (14.0) | 10,999 (61.1) | 79.0 |
| 91-120 days | 18,498 (14.4) | 12,932 (69.9) | 104.0 |
| 121-180 days | 23,155 (18.0) | 17,984 (77.7) | 147.0 |

^a^Six months of treatment is the fixed interval used for calculating the VS proportion in the Brazilian cascade[8].

^b^The Brazilian clinical protocol recommends that the first VL test after ART initiation be performed within eight weeks (56 days).An additional 10 days were added to this period, considering possible administrative difficulties of the facility and/or the patients themselves[29, 38].
