# Supplementary material for: Time from treatment initiation to HIV viral suppression in public care facilities in Brazil: A nationwide linked databases cohort
Source: PLoS One. 2024 Nov 20;19(11):e0305311. doi: 10.1371/journal.pone.0305311 (PMC11578461; doi:10.1371/journal.pone.0305311)
Supplement: S2 Table — Qualiaids-Brazil Cohort, 2015–2018 (N = 84,747). (DOCX) [file pone.0305311.s002.docx]

**S2 Table. Results of hierarchically adjusted multilevel models for time to VS, as well as individual and facility-related characteristics, restricted to those with information on exposure category**. Qualiaids-Brazil Cohort, 2015-2018 (N=84,747).

|  |  |  | **Variable** | **N** | **TR (95% CI)** |
| --- | --- | --- | --- | --- | --- |
|  |  |  |  |  |  |
| **Model 1** | **Model 2** |  | ***Sex*** |  |  |
|  |  |  | Male | 59,479 (70.2%) | ref |
|  |  |  | Female | 25,268 (29.8%) | 0.91[0.90;0.92] |
|  |  |  | ***Age group (in years)*** |  |  |
|  |  |  | 15-19 | 3,978 (4.7%) | 1.13[1.10;1.16] |
|  |  |  | 20-29 | 30,754 (36.3%) | 1.08[1.06;1.10] |
|  |  |  | 30-39 | 24,500 (28.9%) | 1.06[1.04;1.07] |
|  |  |  | 40-49 | 14,787 (17.5%) | 1.04[1.02;1.06] |
|  |  |  | 50-59 | 7,657 (9.0%) | ref |
|  |  |  | ≥60 | 3,071 (3.6%) | 0.98[0.95;1.00] |
|  |  |  | ***Race/skin color*** |  |  |
|  |  |  | White | 39,628 (46.8%) | ref |
|  |  |  | Black | 9,119 (10.8%) | 1.04[1.02;1.05] |
|  |  |  | Yellow | 622 (0.7%) | 1.02[0.97;1.08] |
|  |  |  | Mixed-race | 35,206 (41.5%) | 1.03[1.01;1.04] |
|  |  |  | Indigenous | 172 (0.2%) | 1.15[1.04;1.27] |
|  |  |  | ***Education (schooling years)*** |  |  |
|  |  |  | None | 1,276 (1.5%) | 1.18[1.13;1.22] |
|  |  |  | 1-3 | 7,151 (8.5%) | 1.21[1.19;1.24] |
|  |  |  | 4-7 | 22,384 (26.4%) | 1.15[1.14;1.17] |
|  |  |  | 8-11 | 34,933 (41.2%) | 1.06[1.05;1.07] |
|  |  |  | ≥12 | 19,003 (22.4%) | ref |
|  |  |  | ***Initial therapeutic regimen*** |  |  |
|  |  |  | Preferred regimen (2017-2018: NNRTI+1INI) | 32,405 (38.2%) | ref |
|  |  |  | Preferred regimen (2015-2016: 2NRTI + 1NNRTI) | 51,947 (61.3%) | 1.13[1.12;1.14] |
|  |  |  | Authorized special regimens | 79 (0.1%) | 1.00[0.87;1.16] |
|  |  |  | Unauthorized regimens |  | 1.02[0.95;1.10] |
|  |  |  | ***Initial T-CD4 lymphocyte count (cells/mm³)*** |  |  |
|  |  |  | <200 | 18,955 (22.4%) | 1.09[1.07;1.10] |
|  |  |  | 200-349 | 12,951 (15.3%) | 1.09[1.08;1.11] |
|  |  |  | 350-499 | 19,330 (22.8%) | 1.04[1.03;1.05] |
|  |  |  | ≥500 | 33,511 (39.5%) | ref |
|  |  |  | ***Initial VL count (copies/mL)*** |  |  |
|  |  |  | ≤100,000 | 62,506 (73.8%) | ref |
|  |  |  | >100,000 | 22,241 (26.2%) | 1.18[1.17;1.19] |
|  |  |  | ***Active tuberculosis episode until suppression*** |  |  |
|  |  |  | No | 80,968 (95.5%) | ref |
|  |  |  | Yes | 3,779 (4.5%) | 1.07[1.04;1.09] |
|  |  |  | ***Therapeutic regimen change*** |  |  |
|  |  |  | No | 82,203 (97.0%) | ref |
|  |  |  | Yes | 2,544 (3.0%) | 1.18[1.14;1.21] |
|  |  |  | ***Adherence*** |  |  |
|  |  |  | ≥95% | 36,776 (43.4%) | ref |
|  |  |  | 80-95% | 43,358 (51.2%) | 1.18[1.16;1.19] |
|  |  |  | <80% | 4,613 (5.4%) | 1.74[1.70;1.78] |
|  |  | **Model 3** | ***Geographic region*** |  |  |
|  |  |  | Central-West | 5,402 (6.4%) | 1.16[1.07;1.26] |
|  |  |  | North | 7,737 (9.1%) | 1.16[1.07;1.25] |
|  |  |  | Northeast | 14,799 (17.5%) | 1.19[1.13;1.26] |
|  |  |  | South | 21,508 (25.4%) | 1.03[0.99;1.08] |
|  |  |  | Southeast | 35,301 (41.6%) | ref |
|  |  |  | ***Facility location (municipality)*** |  |  |
|  |  |  | Metropolitan region | 65,875 (77.7%) | ref |
|  |  |  | Other | 18,872 (22.3%) | 1.08[1.04;1.12] |
|  |  |  | ***Number of patients served*** |  |  |
|  |  |  | ≤50 | 2,541 (3.0%) | 1.21[1.13;1.28] |
|  |  |  | 51-500 | 37,125 (43.8%) | 1.03[0.98;1.08] |
|  |  |  | >500 | 45,081 (53.2%) | ref |

Model 1 – sociodemographic characteristics; Model 2 – sociodemographic + clinical characteristics; Model 3 – facility-related characteristics. All models were adjusted for the number of VL tests performed until VS.
