# Supplementary material for: Time from treatment initiation to HIV viral suppression in public care facilities in Brazil: A nationwide linked databases cohort
Source: PLoS One. 2024 Nov 20;19(11):e0305311. doi: 10.1371/journal.pone.0305311 (PMC11578461; doi:10.1371/journal.pone.0305311)
Supplement: S3 Table — Qualiaids-Brazil Cohort, 2015–2018 (N = 84,747). (DOCX) [file pone.0305311.s003.docx]

**S3 Table. Results of hierarchically adjusted multilevel models for time to VS, according to individual and facility-related characteristics WITHOUT adjustment for number of VL tests.** Qualiaids-Brazil Cohort, 2015-2018 (N=84,747).

|  |  |  | **Variable** | **TR (95% CI)** |
| --- | --- | --- | --- | --- |
|  |  |  |  |  |
| **Model 1** | **Model 2** |  | ***Sex*** |  |
|  |  |  | Male | ref |
|  |  |  | Female | 0.91[0.90;0.92] |
|  |  |  | ***Age group (in years)*** |  |
|  |  |  | 15-19 | 1.08[1.06;1.11] |
|  |  |  | 20-29 | 1.04[1.03;1.06] |
|  |  |  | 30-39 | 1.06[1.04;1.08] |
|  |  |  | 40-49 | 1.04[1.02;1.06] |
|  |  |  | 50-59 | Ref |
|  |  |  | ≥60 | 0.96[0.94;0.99] |
|  |  |  | ***Race/skin color*** |  |
|  |  |  | White | ref |
|  |  |  | Black | 1.02[1.04;1.04] |
|  |  |  | Yellow | 1.02[0.97;1.08] |
|  |  |  | Mixed-race | 1.02[1.01;1.03] |
|  |  |  | Indigenous | 1.20[1.08;1.32] |
|  |  |  | ***Education (in years of schooling)*** |  |
|  |  |  | None | 1.23[1.19;1.27] |
|  |  |  | 1-3 | 1.25[1.23;1.28] |
|  |  |  | 4-7 | 1.20[1.18;1.22] |
|  |  |  | 8-11 | 1.07[1.06;1.09] |
|  |  |  | ≥12 | ref |
|  |  |  | ***Initial therapeutic regimen*** |  |
|  |  |  | Preferred regimen (2017-2018: NNRTI+1INI) | ref |
|  |  |  | Preferred regimen (2015-2016: 2NRTI + 1NNRTI) | 1.13[1.13;1.15] |
|  |  |  | Authorized special regimens | 1.03[0.91;1.16] |
|  |  |  | Unauthorized regimens | 1.03[0.97;1.09] |
|  |  |  | ***Initial T-CD4 lymphocyte count (cells/mm³)*** |  |
|  |  |  | <200 | 1.09[1.08;1.11] |
|  |  |  | 200-349 | 1.08[1.07;1.09] |
|  |  |  | 350-499 | 1.04[1.03;1.05] |
|  |  |  | ≥500 | ref |
|  |  |  | ***Initial VL count (copies/mL)*** |  |
|  |  |  | ≤100,000 | ref |
|  |  |  | >100,000 | 1.17[1.16;1.18] |
|  |  |  | ***Active tuberculosis episode until suppression*** |  |
|  |  |  | No | ref |
|  |  |  | Yes | 1.06[1.04;1.08] |
|  |  |  | ***Therapeutic regimen change*** |  |
|  |  |  | No | ref |
|  |  |  | Yes | 1.15[1.13;1.17] |
|  |  |  | ***Adherence*** |  |
|  |  |  | ≥95% | ref |
|  |  |  | 80-95% | 1.19[1.18;1.20] |
|  |  |  | <80% | 1.81[1.78;1.85] |
|  |  | **Model 3** | ***Geographic region*** |  |
|  |  |  | Central-West | 1.13[1.06;1.21] |
|  |  |  | North | 1.17[1.09;1.24] |
|  |  |  | Northeast | 1.17[1.12;1.22] |
|  |  |  | South | 1.03[0.99;1.07] |
|  |  |  | Southeast | ref |
|  |  |  | ***Facility location (municipality)*** |  |
|  |  |  | Metropolitan region | ref |
|  |  |  | Other | 1.06[1.03;1.10] |
|  |  |  | ***Number of patients served*** |  |
|  |  |  | ≤50 | 1.19[1.13;1.25] |
|  |  |  | 51-500 | 1.03[0.98;1.07] |
|  |  |  | >500 | ref |

Model 1 – sociodemographic characteristics; Model 2 – sociodemographic + clinical characteristics; Model 3 – facility-related characteristics. The models were not adjusted for the number of VL tests performed until VS[79].
